# Comparative Evaluation of Oral Health Status and Treatment Needs of Children With Autism Spectrum Disorder: A Cross-Sectional Study

**DOI:** 10.7759/cureus.58663

**Published:** 2024-04-20

**Authors:** Vashi Narula, Mridula Goswami, Monica Juneja, Gyanendra Kumar

**Affiliations:** 1 Pediatric and Preventive Dentistry, Maulana Azad Institute of Dental Sciences, New Delhi, IND; 2 Pediatrics, Lok Nayak Hospital, New Delhi, IND

**Keywords:** treatment needs, oral hygiene status, dental caries, oral health, autism spectrum disorder

## Abstract

Introduction:* *Oral health care is essential yet challenging in children with autism spectrum disorder (ASD) due to their impaired emotional and behavioral stability, lack of communication skills, and inability to perform daily home hygiene routines properly. The present study was planned with the aim of assessing the Oral Health Status and Treatment Needs of children with ASD in comparison with children without any systemic disease.

Methods: A total of 160 children, in the age group of 5-14 years, divided equally into two groups, i.e., Group A (children with ASD) and Group B (children without any systemic disease), were assessed for Dental caries, Oral Hygiene Status, and Treatment Needs. The behavior of children in each group, during oral examination, was also assessed and recorded. Student t test and Chi square test were used for quantitative and qualitative analysis, respectively.

Results: The mean age of participating children was 7.96±2.43 years with a male predominance (male to female ratio - 1.58:1). Children with ASD displayed more negative behavior with 15% showing definitely negative behavior, 21.2% negative behavior on Frankl’s Behavior Rating scale; the statistically lower prevalence of dental caries (Group A - mean decayed, missing and filled primary teeth (dmft): 1.7±3.2, mean decayed, missing and filled permanent teeth (DMFT): 0.19±0.71; Group B - mean DMFT: 5.44±4.88, mean DMFT: 1.01±1.51; p=0.0001), better oral hygiene (Group A - 18.8% showed good Simplified Oral Hygiene Index Score (OHI-S), 56.2% showed fair OHI-S; Group B - 6.2% showed good OHI-S and 46.3% showed fair OHI-S) and lower treatment needs compared to children without any systemic disease.

Conclusion:* *Children with ASD showed better oral health and lower treatment needs. This suggests that introducing oral hygiene care and diet modifications in daily routine can significantly improve the Oral Health Status in children with ASD.

## Introduction

Oral health care and maintenance is an essential component of overall growth and development of children. Inculcating and following good oral hygiene practices with appropriate diet modification is a prerequisite to achieving sound oral health [1]. Performing oral hygiene techniques and utilizing oral hygiene aids require basic learning and understanding skills as well as gross motor skills. This is a major obstacle in children with Special Health Care Needs (SHCN) lacking individual capability of self-care [2]. Children with Autism Spectrum Disorder (ASD) tend to lack psychomotor control and the ability to understand, follow, or cooperate with preventive oral health practices. The present literature reports that children with ASD generally exhibit an overall poor oral hygiene status possibly due to impaired manual dexterity, emotional or behavioral instability, and inability to perform daily home hygiene routine properly [3-5]. Therefore, there is a need to assess the Oral Health Status and Treatment Needs of children with ASD to evaluate the overall dental disease burden in these children. This assessment is also essential to enhance the provision of preventive and therapeutic dental services to children with ASD. It also forms baseline data for the development of various Oral Health Programs and Strategies to increase knowledge and awareness on oral health care and maintenance among parents/guardians as well as children with autistic traits. Hence, this study was planned to assess the Oral Health Status and Treatment Needs of children with ASD in comparison with children without any systemic disease.

## Materials and methods

Ethical approval

The present cross-sectional study was conducted after obtaining approval from the Ethical Committee of the Institution (Ref no.- MAIDS/Ethical Committee/3541) with written informed consent from parents/guardians of all participants.

Study population

The study sample comprised 160 children which were divided equally into two groups. Group A included 80 children pre-diagnosed with ASD, between the age group of 5-14 years, at the Child Development Centre, Lok Nayak Hospital, New Delhi. Children with any other additional symptom/disorder/disease known to be associated with ASD were excluded. Group B included 80 children, without any systemic disease, attending the O. P. D. of Pediatric and Preventive Dentistry, Maulana Azad Institute of Dental Sciences, New Delhi. Children with any medical disease, physical disability, or mental illness were excluded. The participants in each group were matched randomly in terms of age and sex to eliminate confounding.

Tools for data collection

Children in both groups were assessed for their behavior during oral examination using Frankl’s Behavior Rating Scale (FBRS) [6]. Each participant was scored by a single investigator on their behavior during a dental check-up. Children were rated from 1-4 for the display of definitely negative, negative, positive, and definitely positive behavior in order. 

**Figure 1:**
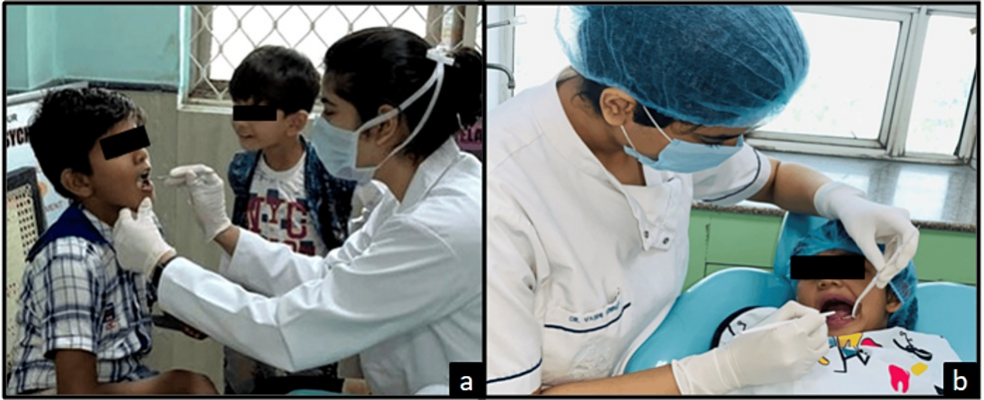
(a) Assessment of oral indices in Group A, (b) assessment of oral indices in Group B Group A - children with Autism Spectrum Disorder, Group B - children without any systemic disease

Assessment of oral health status was done using oral hygiene and dental caries assessment tools. The Oral Hygiene Status of each participant was evaluated using the Simplified Oral Hygiene Index OHI-S [7] and Plaque Index PI [8]. OHI-S was used to detect the presence of debris and calculus in all the children (Figures 1a, 1b). The presence of debris, calculus, and plaque was assessed using a disposable plain mouth mirror and explorer. The estimation of debris using Debris Index-Simplified (DI-S) was done prior to the estimation of calculus using Calculus Index-Simplified (CI-S). Six index teeth (16,11,26,36,31,46 for permanent dentition and 55,51,65,75,71,85 for primary dentition) were assessed and scored based on the index criteria (Table 1). A DI-S and CI-S score is obtained by the sum of individual scores of all index teeth divided by the total number of teeth assessed for each child. The final OHI-S score for each participant was calculated by the sum of their DI-S and CI-S scores (Table 2).

**Table 1:** Scoring criteria for assessment of oral hygiene DI-S - Simplified Debris Index, CI-S - Simplified Calculus Index; Dental Index - Simplified Oral Hygiene Index by Greene Vermillion, 1964 [7]

Simplified Debris Index (DI-S) Scoring Criteria	
Score	Criteria
0	No debris or stain present
1	Soft debris covering not more than one third of tooth surface, or presence of extrinsic stains without other debris regardless of surface area covered
2	Soft debris covering more than one third, but not more than two thirds, of the exposed tooth surface
3	Soft debris covering more than two thirds of the exposed tooth surface
Simplified Calculus Index (CI-S) Scoring Criteria	
Score	Criteria
0	No calculus present
1	Supragingival calculus covering not more than one third of tooth surface
2	Supragingival calculus covering more than one third, but not more than two thirds, of the exposed tooth surface or presence of individual flecks of subgingival calculus around the cervical portion of the tooth or both.
3	Supragingival calculus covering more than two thirds of the exposed tooth surface or a band of subgingival calculus around the cervical portion of the tooth or both

**Table 2:** Calculation and Interpretation of OHI-S OHI-S - Simplified Oral Hygiene Index; Dental Index - Simplified Oral Hygiene Index by Greene and Vermillion, 1964 [7]

Interpretation	DI-S	CI-S	OHI-S= DI-S+CI-S
Good	0.0-0.6	0.0-0.6	0.0-1.2
Fair	0.7-1.8	0.7-1.8	1.3-3.0
Poor	1.9-3.0	1.9-3.0	3.1-6.0

For evaluating the Plaque Index (PI) four surfaces (Disto-facial, Facial, Mesio-facial, and lingual) of six indexed teeth (16,12,24,36,32,44 for permanent dentition and 55,52,64,75,72,84 for primary dentition) were assessed and scored based on the quantity of plaque as per index criteria (Table 3). PI was then calculated by dividing the sum of the scores of each surface divided by the total number of surfaces assessed (Table 4).

**Table 3:** Scoring Criteria for assessment of plaque Dental Index - Plaque Index (PI) by Silness and Loe, 1964 [8]

Score criteria for each tooth surface	
Score	Criteria
0	No Plaque
1	A film of plaque adhering to the free gingival margin and adjacent area of tooth. The plaque may be seen only by running a probe across the tooth surface.
2	Moderate accumulation of soft deposits within the gingival pocket, on the gingival margin and/or adjacent tooth surface, which can be seen by naked eye.
3	Abundance of soft matter within gingival pocket and /or on the gingival margin and adjacent tooth surface.

**Table 4:** Interpretation of plaque index PI - Plaque Index; Dental Index - Plaque Index by Silness and Loe, 1964 [8]

Interpretation	Score
Excellent	0
Good	0.1-0.9
Fair	1.0-1.9
Poor	2.0-3.0

Assessment of Dental caries was done using WHO oral health assessment criteria for children, 2013 [9]. The number of decayed, missing, and filled permanent teeth (DMFT) was recorded as well as decayed, missing, and filled primary teeth (dmft). Evaluation of dental treatment needs was again done using the WHO oral health assessment criteria for children, 2013. Children were scored based on the interventional urgency from 0 to 4 wherein each progressing score reflected the increased urgency of treatment.

The level of severity of ASD was already pre-recorded by associated medical practitioners based on the Childhood Autism Rating Scale-2 (CARS-2) [10] for each participant in Group A from mild to moderate and severe.

Statistical analysis

The collected data was recorded in Microsoft Excel v365. Data analysis was performed using Statistical Package for Social Sciences software (IBM SPSS v23.0; IBM Corp., Armonk, NY). Descriptive statistics including mean, standard deviation, and percentages were calculated for each variable. Significance for all statistical tests was predetermined at a probability value less than 0.05. The chi-square test was used to analyze qualitative variables and the student t-test to assess quantitative variables. The Pearson Correlation test was used to determine the correlation coefficient.

## Results

In the present study, the mean age of children in Group A (children with ASD) and Group B (children without any systemic disease) was 7.96±2.43 years. The participating children were also matched in terms of sex for the elimination of confounding. In Group A and Group B, 49 (61.2%) children were males, and 31 (38.8%) children were females.

Based on the educational status, 7.5% fathers of children in Group A were uneducated, 46.2% had completed their schooling, 37.5% were graduates and 8.8% had completed their post-graduation. In Group B, 27.4% fathers of children participating in the study had studied in school 63.8% were graduates and 8.8% fathers had completed their post-graduation. Out of a sample of 80, 16.2% mothers of children in Group A were uneducated, 52.6% had studied and 21.2% were graduates, and 10% mothers had completed their post-graduation. In Group B, 2.5% mothers of children participating in the study were uneducated, 38.7% mothers had studied in school, 55% mothers were graduates and 3.8% had completed their post-graduation.

**Figure 2:**
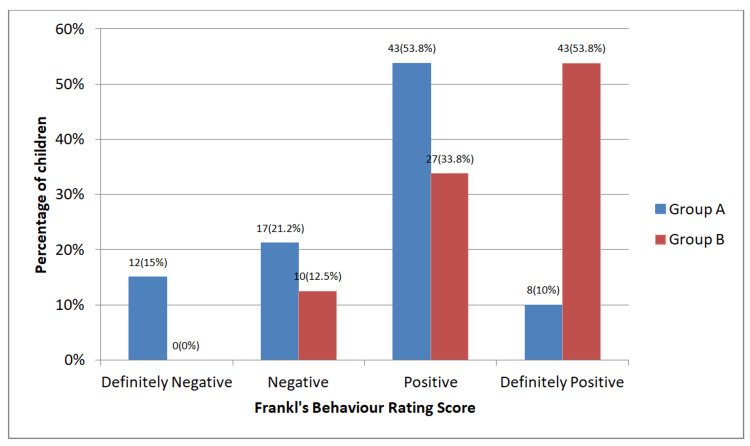
Frankl’s Behaviour Rating of participating Children Group A- children with Autism Spectrum Disorder, Group B- children without any systemic disease; p value=0.54 (statistically not significant); statistical analysis- chi square test

Based on behavior assessment during the oral examination, in Group A, 15% children with ASD showed definitely negative behavior on Frankl’s Behavior Rating scale, 21.2% children showed negative behavior, 53.8% children showed positive behavior and 10% children showed definitely positive behavior. In Group B, no child showed definitely negative behavior on Frankl’s Behavior Rating scale, 12.5% children showed negative behavior, 33.8% children showed positive behavior and 53.8% children showed definitely positive behavior (Figure 2).

Assessing the Dental Caries Status, children with ASD in Group A showed a mean DMFT score of 1.7±3.2 with the standard error of mean 0.36. In Group B, the mean DMFT score of children was 5.44±4.88 with the standard error of the mean being 0.55. The mean DMFT score of Group A was 0.19±0.71 with a standard error of the mean of 0.08. The mean DMFT score of Group B was 1.01±1.51 with a standard error of the mean of 0.17. The comparison of dental caries status in primary and permanent dentition between Group A and Group B showed statistically significant results (p=0.0001) (Table 5).

**Table 5:** Mean dmft/DMFT score of children in Group A and Group B Group A - Children with Autism Spectrum Disorder, Group B - Children without any systemic disease; dmft - decayed, missing and filled primary teeth; DMFT - Decayed, Missing and Filled Permanent Teeth; statistical analysis - student t-test

Group	(N)	dmft		DMFT	
		Mean±SD	Std. error of mean	Mean±SD	Std. error of mean
Group A	80	1.7±3.2	0.36	0.19±0.71	0.08
Group B	80	5.44±4.88	0.55	1.01±1.51	0.17
Level of Significance		0.0001 (Group B>Group A)		0.0001 (Group B>Group A)	

**Figure 3:**
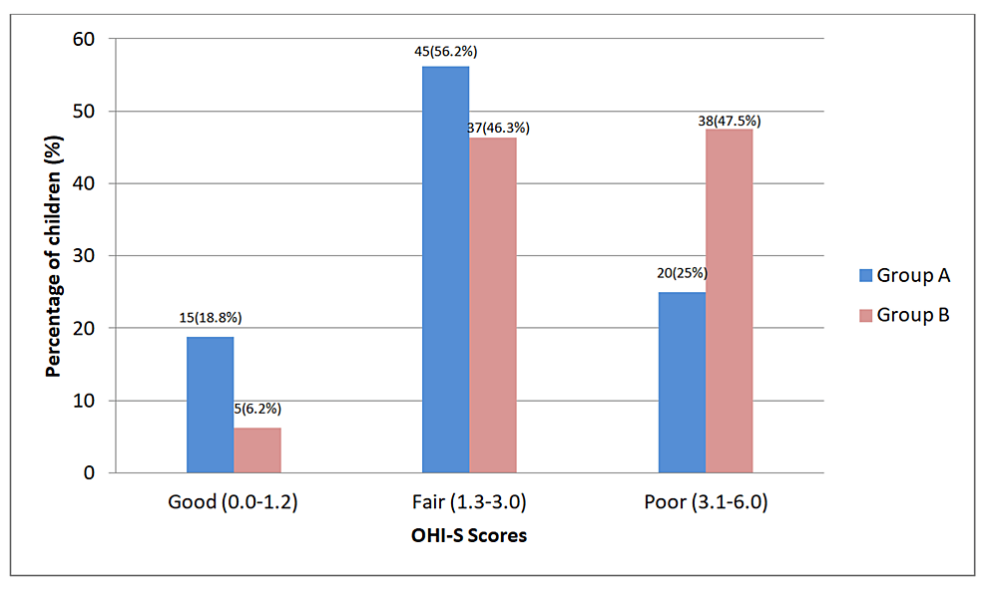
Percentage distribution of OHI-S scores of Group A and Group B Group A - children with Autism Spectrum Disorder, Group B - children without any systemic disease; p-value=0.001 (statistically significant); statistical analysis - student t-test

Based on the Oral Hygiene Status evaluation, the Simplified Oral Hygiene Index (OHI-S) scores of children in Group A showed that 18.8% children had a good OHI-S score, 56.2% had a fair OHI-S score and 25% had a poor OHI-S score. In Group B, 6.2% children had a good OHI-S score, 46.3% had a fair OHI-S score and 47.5% had a poor OHI-S score (Figure 3).

**Figure 4:**
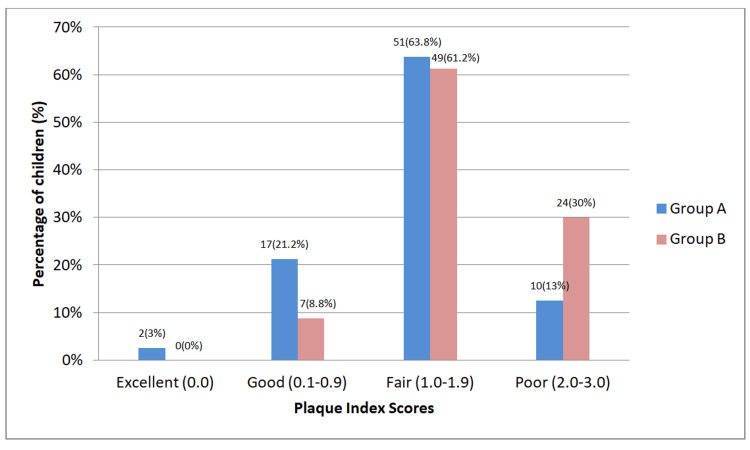
Percentage distribution of Plaque Index scores of Group A and Group B Group A - children with Autism Spectrum Disorder, Group B - children without any systemic disease; p-value=0.023 (statistically significant); statistical analysis - student t-test

The Plaque index scores of children in Group A showed that 3% children had an excellent Plaque index score, 21.2% had a good Plaque index score, 63.8% had a fair Plaque index score and 12.5 had a poor Plaque index score. In Group B, 8.8% children had a good Plaque index score, 61.2% had a fair Plaque index score and 30% had a poor Plaque index score (Figure 4).

The Treatment Needs of children in Group A showed that 10% children did not require any dental treatment, 38.8% children required preventive or routine dental treatment, 48.8% children required prompt dental treatment including scaling, and 2.5% children required immediate (urgent) treatment due to pain or dental infection. In Group B, 2.5% children required preventive or routine dental treatment, 5% children required prompt dental treatment including scaling and 92.5% children required immediate (urgent) treatment due to pain or dental infection (Table 6).

**Table 6:** Dental treatment needs of Group A and Group B Group A - Children with Autism Spectrum Disorder, Group B - Children without any systemic disease

Treatment Needs	Groups			
	Group A		Group B	
	Frequency (n)	Percentage (%)	Frequency (n)	Percentage (%)
No treatment needed	8	10.0	0	0.0
Preventive or routine treatment needed	31	38.8	2	2.5
Prompt treatment needed	39	48.8	4	5.0
Immediate (urgent) treatment needed	2	2.5	74	92.5
Referred for comprehensive evaluation (systemic)	0	0.0	0	0.0
Total	80	100.0	80	100.0

Based on the medical records of children with ASD (Group A), the severity of symptoms was recorded using the Childhood Autism Rating Scale-2 (CARS-2). The majority of the children assessed showed Mild to Moderate symptoms of ASD with a mean score of 32.29±3.48. The correlation of the CARS-2 scores with the Oral Health Status of children with ASD in Group A showed a statistically significant relation between the CARS-2 score and OHI-S (p-value= 0.004) and Plaque Index score (p value= 0.04) of these children. This suggests that with an increase in the severity of symptoms of ASD, the oral hygiene of children decreased significantly (Table 7).

**Table 7:** Correlation between CARS-2 score and oral health status of children with ASD CARS-2 - Childhood Autism Rating Scale; DMFT - Decayed, Missing and Filled Permanent Teeth; dmft - decayed, missing and filled primary teeth; OHI-S - Simplified Oral Hygiene Index; PI - Plaque Index

CARS-2 Score	DMFT	dmft	OHI-S	PI score
Pearson Correlation	-0.104	-0.019	0.316	0.230
p value	0.360	0.864	0.004	0.04

## Discussion

ASD is a special health condition diagnosed at a young age that requires mild to substantial functional support throughout life due to inherent neuro-developmental impairment [11]. Loo et al. [12] and Marshall et al. [13] found that oral health challenges among children with ASD are high and increase exponentially with the level of severity of symptoms, dependency on the caregiver for basic life skills, advancing age, and, associated pathologies. A paucity of information is seen regarding the evaluation of oral health in children with ASD in India with no study assessing the Oral Health Status in North India. Hence, the present study assessed the Oral Health Status and Treatment Needs of children with ASD and compared the parameters with data collected on similar characteristics in children without any systemic disease. 

The age group of children included in the present study was between 5 and 14 years. The mean age of children with ASD (Group A) matched with children without any systemic disease (Group B) was 7.96±2.43 years with a maximum of children with age five years. According to the American Academy of Pediatrics, the diagnostic signs of ASD appear as early as 24 months of age [14]. McGovern and Sigman [15] reported that most children were diagnosed with ASD by the age of 2-5 years. Most of the symptoms of ASD manifest completely by five years of age. Hence, children of this age report more frequently to the development center for screening and diagnosis of ASD.

In the present study, sex distribution in each group showed that 61.2% of the sample comprised males and 38.8% were females. The male-to-female ratio was 1.58. WHO reported similar findings in an ASD awareness meeting with a high male-to-female ratio of 4:1 [16]. Similar results were reported in a systematic review by Loomes et al. [17] who stated an ASD male-to-female ratio of 3:1. A striking male bias has been seen in the prevalence of ASD indicating the involvement of sex-specific biological factors in ASD etiology but no definitive explanation has yet been found on this finding [18].

In the current study, the demographics on the educational level of parents revealed that 16.2% mothers of children in Group A were uneducated, 22.5% had completed their schooling and 21.2% were graduates. In Group B, only 2.5% mothers of the participants were uneducated while 55% were university graduates. This showed that a higher number of mothers of children with ASD were found to be uneducated as compared to children without any systemic disease. Lung et al. [19] reported similar results stating that mothers with lower than average educational levels were associated with children being at high risk of ASD but mothers with higher than average educational levels reported to the testing center more often.

Children with ASD lack cognitive abilities, communication skills, and adaptive behaviors and pose a significant challenge in oral assessment and treatment [20]. Various scales are used to assess behavior, particularly dental fear, in children. Frankl’s Behavior Rating Scale is the most widely used scale to measure and classify child behavior [21]. In the present study, in Group A, 21.2% showed negative behavior and 15% showed definitely negative behavior during the oral examination. In Group B, 53.8% children showed definitely positive behavior and 33.8% showed positive behavior during an oral examination. This shows that children without any systemic disease showed more positive behavior than children with ASD. Stein et al. [22] stated that several research indicate that children with ASD exhibit more dental behavior management problems compared to typically developing children with approximate number of 50%-72% children with ASD exhibiting uncooperative behavior in a dental clinic [23]. El Khatib et al. [24] also found that more children with ASD behaved “negatively” or “definitely negatively” (37% and 11%) than healthy controls (11% and 2%) during dental treatment. In the present study, more children with ASD showed an intra-group positive behavior. This could be due to the relatively safe surroundings provided during the dental examination which avoided the use of a fear-provoking dental chair unit and using a simple dental procedure such as oral examination and indices evaluation. Dangulavanich et al. [25] also found that children with ASD undergoing oral examination displayed a more cooperative behavior (62.96%) as compared to those undergoing therapeutic dental procedures (27.78%).

**Figure 5:**
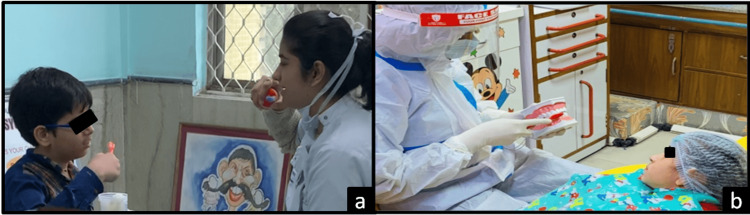
(a) Oral hygiene instructions in Group A, (b) oral hygiene instructions in Group B Group A - children with autism spectrum disorder, Group B - children without any systemic disease

In the present study, the mean dmft score for primary dentition in Group A was 1.7±3.2 compared to 5.44±4.88 in Group B. The findings suggested that children with ASD exhibited a lower prevalence of dental caries in the primary dentition, in the current sample, as compared to children without any systemic disease. The result was statistically significant (p=0.0001) with a mean difference of 0.825. Kuter & Guler (2019) [26], Hussein et al. [27], Du et al. [3], and Vajawat and Deepika [4] reported similar findings. Richa et al. [28] and Jaber [29] reported contradictory results showing a higher prevalence of dental caries in primary teeth in children with ASD compared to healthy children without ASD. The present study results also showed a lower prevalence of DMFT in permanent dentition in Group A with a mean DMFT score of 0.19±0.71 compared to Group B with a mean DMFT score of 1.01±1.51. The results were statistically significant (p=0.0001) with a mean difference of 3.738. These results were in accordance with research by Kuter and Guler [26] and Hussein et al. [27]. In contrast to the above results, Richa et al. [28] and Jaber (2011) [29] reported a higher prevalence of dental caries in permanent dentition in children with ASD compared to healthy children without ASD. Children with ASD were given a detailed oral care routine by the special educator at the development center. This included oral hygiene instructions such as tooth brushing and mouth rinsing with diet modifications having less sugar intake to reduce uncontrolled aggressive behaviors. This routine practice rehearsed at every visit resulted in lesser dental caries prevalence in children receiving therapy from the Child Development Center. Furthermore, the majority of children at the center displayed mild to moderate symptoms of ASD with characteristic parrot speech and adequate understanding. Hence, they were able to follow the prescribed oral hygiene routine religiously under the guidance of their caregivers. Children in Group B, however, did not comply well with the oral hygiene instructions (Figures 5a, 5b). Therefore, comparatively higher dental caries prevalence can be seen in these children. Children in both groups exhibited higher caries prevalence in primary teeth than in permanent teeth. This is in accordance with a study by Morales-Chavez [30] who reported a higher prevalence of dental caries in primary teeth as compared to permanent teeth with a ratio of 1:2.5.

In the present study, Oral Hygiene Status was evaluated using the Simplified Oral Hygiene Index (OHI-S) and Plaque Index (PI). In Group A, 56.2% children had fair OHI-S scores and 25% had poor OHI-S scores. In Group B, 46.3% had a fair OHI-S scores and 47.5% had poor OHI-S scores. This suggests that the majority of children with ASD had fair OHI-S scores compared to children without any systemic disease, who had a poorer OHI-S score. This could be due to the daycare routine prescribed by special educators to children with ASD at CDC which included tooth brushing, mouth rinsing, and low sugar diet. Hussein et al. [27], in a study on the assessment of periodontal health status in a sample of autistic children, stated that both the test and control groups showed fair OHI-S scores with autistic children showing less calculus compared to unaffected children. Morales-Chavez [30] contrastingly reported a mean OHI-S score of 3.4, coinciding with the poor oral hygiene status, in children with ASD. They also reported that 59.41% children with ASD showed the presence of calculus [29]. Richa et al. [28] also reported contrasting results with a poor OHI-S score in children with ASD in comparison to a good OHI-S score in unaffected children. A high mean OHI-S score showing fair to poor oral hygiene status in children with ASD could be due to irregular brushing habits and difficulties encountered by parents and caregivers in brushing their child’s teeth.

In the current study, 63.8% children in Group A had a fair Plaque Index (PI) score with a mean PI score of 1.34±0.59. In Group B, 61.2% children had a fair PI score with a mean PI score of 1.69±0.48. The result was statistically significant (p value=0.023). Children in both groups had fair plaque presence with children without systemic disease having more plaque compared to children with ASD. Similar results were shown by Du et al. [3] and Vajawat and Deepika [4] who also reported fair mean PI scores in both test and control groups. Contrastingly, Bossu et al. [5] reported that 60% children with ASD showed poor periodontal health with abundant plaque. Kuter and Guler [26] also reported a generalized poor oral hygiene status in the participating children with a mean PI score of 2.6±0.48 in children with ASD and 2.66±0.56 in children without autism. The abundance of plaque in children with ASD can lead to marginal gingivitis. This could be due to the lack of proper understanding and manual dexterity in maintaining oral hygiene by children with ASD.

In Group A, 48.8% children required prompt treatment including oral prophylaxis, and 38.8% required preventive or routine treatment. In Group B, 92.5% required immediate (urgent) treatment. A high proportion of children report to the OPD at MAIDS because of acute dental pain or chronic dental problems, requiring immediate treatment. Hence, a higher percentage of urgent treatment needs is seen in Group B children. In a similar study, Jaber [29] also reported that 100% children with ASD required oral prophylaxis compared to 41% children without.

This cross-sectional study showed that repeated oral hygiene instructions during medical visits of children with ASD significantly improved their oral health status than those without. The limitations of the present study include the assessment of autistic children with adequate oral hygiene guidance at every medical visit resulting in contrasting results. Therefore, further research is required to assess the oral health status of a larger sample of children with ASD in the general population without any such regular guidance.

## Conclusions

Children with ASD showed poor behavior control during their oral examination. Hence, higher emphasis needs to be given to behavior management of these children which proves to be extremely challenging while rendering dental treatment. In the present study children with ASD were guided well to follow a daily routine plan as prescribed by the special educators. This regimen proved helpful in improving the Oral Health condition of these children effectively. This reinforces the idea of collaboration of pediatric dental clinics with medical facilities to impart Oral Health Education and training to children with ASD at their primary care center. As a result, a comprehensive, specially designed Oral Health Education Program for children with ASD and their caregivers needs to be formulated and implemented. The present study also raises the need for further research in this field. Assessment of the Oral Health Status and Treatment Needs of a larger sample of children with ASD can help in an accurate estimation of the oral disease burden of these children as a representative of the entire population.
